# Change in Enlarged Perivascular Spaces over Time and Associations with Outcomes After Traumatic Brain Injury

**DOI:** 10.1089/neur.2024.0026

**Published:** 2024-07-31

**Authors:** Alexa E. Walter, Krupa Savalia, Jason Yoon, Justin Morrison, Andrea L. C. Schneider, Ramon Diaz-Arrastia, Danielle K. Sandsmark

**Affiliations:** ^1^ Department of Neurology, University of Pennsylvania Perelman School of Medicine, Philadelphia, Pennsylvania, USA.; ^2^ Departments of Neurology and Neurological Surgery, University of California Davis, Davis, California, USA.; ^3^ Department of Neurology, Beth Israel Deaconess Medical Center, Harvard Medical School, Boston, Massachusetts, USA.; ^4^ Departments of Neurology and Biostatistics, Epidemiology, and Informatics, University of Pennsylvania Perelman School of Medicine, Philadelphia, Pennsylvania, USA.

**Keywords:** enlarged perivascular spaces, magnetic resonance imaging, perivascular spaces, traumatic brain injury

## Abstract

Enlarged perivascular spaces (EPVs) can be seen on magnetic resonance imaging (MRI) scans in various neurological diseases, including traumatic brain injury (TBI). EPVs have been associated with cognitive dysfunction and sleep disturbances; however, their clinical significance remains unclear. The goal of this study was to identify MRI burden of EPVs over time following TBI and to explore their relationship with postinjury outcomes. Individuals with TBI underwent postinjury data collection at Day 1 (blood), 2 weeks (blood, MRI, outcomes), and 6 months (blood, MRI, outcomes). EPV burden was assessed using T1 and FLAIR sequences on representative slices in the centrum semiovale, basal ganglia, and midbrain. Serum blood was assayed to measure concentrations of neurofilament light (NfL) and glial fibrillary acidic protein (GFAP). Thirty-two participants with TBI were included (mean age 36.8 years, 78% male, 50% White). Total EPVs count did not significantly change from 2 weeks (23.5 [95% confidence interval or CI = 22.0–32.0]) to 6 months (26.0 [95% CI = 22.0–30.0], *p* = 0.16). For self-reported measures of sleep, there were no significant associations between EPVs count and Insomnia Severity Index (2 weeks: β = −0.004; 95% CI = −0.094, 0.086; 6 months: β = 0.002; 95% CI = −0.122, 0.125) or the subset of sleep questions on the Rivermead Post-Concussion Symptoms Questionnaire (2 weeks: β = −0.005; 95% CI = −0.049, 0.039; 6 months: β = −0.019; 95% CI = −0.079, 0.042). Functional outcome, determined by 6 months incomplete recovery (Glasgow Outcome Scale-Extended [GOS-E < 8]) versus complete recovery (GOS-E = 8), was significantly associated with a higher number of EPVs at 2 weeks (odds ratio = 0.94, 95% CI = 0.88–0.99). Spearman correlations showed no significant relationship between EPVs count and GFAP or NfL. This study used commonly acquired MRI sequences to quantify EPVs and investigated their utility as a potential imaging biomarker in TBI. Given the minimal change in EPVs over time, this period may not be long enough for potential recovery or may indicate that EPVs are structural findings that do not significantly change over time.

## Introduction

Perivascular spaces, also known as Virchow–Robin spaces,^1^ are fluid-filled regions surrounding brain arterioles, venules, and capillaries. These spaces are thought to be involved in maintaining brain homeostasis and facilitating the removal of metabolites from the brain interstitial fluid, particularly during sleep.^2,3^ These spaces can become enlarged (enlarged perivascular spaces; EPVs), a finding of unclear significance that has long been appreciated on brain magnetic resonance imaging (MRI) scans. However, recent work has linked EPVs to aging,^4,5^ cognitive impairment,^6^ Parkinson’s disease,^7^ multiple sclerosis,^8^ Alzheimer’s disease,^9^ and stroke.^10,11^

Although the presence of EPVs is common among individuals with neurological diseases, the role that they play in the pathophysiology of these various central nervous system disorders is still uncertain. Hypotheses include that EPVs represent impaired interstitial fluid drainage or glymphatic system dysregulation, although strong supportive evidence is lacking.^12,13^ Glymphatic dysregulation has also been shown to be disrupted after traumatic brain injury (TBI) and may be linked with postinjury sleep deficits.^13,14^ Studies have shown greater EPVs burden in TBI.^12,15,16^ However, findings with sleep have been mixed with some studies showing that increased EPVs burden is associated with sleep deficits^12,15^ and others showing no relationship.^16^

There is much still not understood about EPVs in TBI, including their evolution over time postinjury or their link to other markers of injury, including blood-based biomarkers. Currently, there is limited ability to categorize TBI as well as predict long-term outcomes postinjury. However, blood-based biomarkers have been offering promise as both diagnostic and prognostic tools. Given the potential relationship between EPVs, sleep, and the glymphatic system, it is possible that blood-based biomarkers may provide additional insights into the underlying pathology given the role of the glymphatic system in transporting these biomarkers.^17^ Glial fibrillary acidic protein (GFAP), in particular, has been hypothesized to be an indicator of glymphatic dysfunction given the role of astrocytes in the neurovascular unit,^18^ as well as its drainage via interstitial space to the blood by the glymphatic system.^17^

Therefore, the overall goal of this study was to use a previously validated technique^5,19^ to evaluate the presence of EPVs in individuals who had sustained a traumatic brain injury (TBI). Objectives of this study were to (1) identify the MRI burden of EPVs over time following TBI from 2 weeks to 6 months postinjury, (2) identify the relationship between EPVs and postinjury sleep-related outcomes, (3) explore the relationship between EPVs and functional outcome and measures of self-reported symptomatology, and (4) identify the relationship between EPVs and blood-based biomarkers.

## Methods

### Participants

Participants were enrolled in an observational cohort study that included individuals with mild-to-severe head injuries. Included participants were 18–65 years old with a TBI diagnosis who were enrolled within 72 h of arriving at the University of Pennsylvania’s Level I Trauma Center. Inclusion criteria also included a clinical diagnosis of TBI, based on the American Congress of Rehabilitation Medicine criteria,^20^ and the ability to undergo an MRI. Participants were excluded based on (a) history of disabling preexisting neurological disease, (b) history of premorbid debilitating condition that interfered with outcome assessments, (c) bilaterally absent pupillary responses, (d) penetrating TBI, (e) requirement of craniotomy or craniectomy, (f) midline shift >3 mm at the level of septum pellucidum or any focal or high-density lesion >10 mL in volume, (g) elevated intracranial pressure, (h) history of prior hospitalization for TBI >1 day, (i) contraindication to MRI, (j) prisoners or patients in police custody, or (k) pregnancy. After eligibility was determined, competence to provide consent was assessed through the Galveston Orientation and Amnesia Test (GOAT).^21^ A GOAT score of ≥75 was considered competent, and consent was obtained from the patient. If the patient scored <75, or was unable to participate in the GOAT assessment, consent was obtained from a legal authorized representative. All study procedures were approved by the University of Pennsylvania’s Institutional Review Board and were done in accordance with the Declaration of Helsinki.

### Study timepoints

Demographic, medical history, injury characteristics, and a blood draw were collected at the time of enrollment. Once screening and eligibility were assessed, participants underwent a battery of testing, including MRI scans, a blood draw, and outcome measures completed at 2 weeks and 6 months postinjury. Only participants with paired MRI at 2 weeks and 6 months postinjury were included in analyses.

### MRI scans

All scans were run on a 3T Siemens PrismaFit (Siemens, Erlangen, Germany) using a 32-channel head coil. Identical imaging protocols were obtained at 2 weeks and 6 months postinjury and included the following: T1 MPRAGE (acquisition time (TA) = 5:12, resolution = 1.0 × 1.0 × 1.0 mm, repetition time (TR) = 2300 ms, echo time (TE) = 2.94 ms, flip angle = 9°, slices = 208); FLAIR 3D T2 Turbo (TA = 6:44, resolution = 0.5 × 0.5 × 1.2 mm, TR = 6000 ms, TE = 390 ms, slices = 176).

### EPVs categorization

**FIG. 1.**
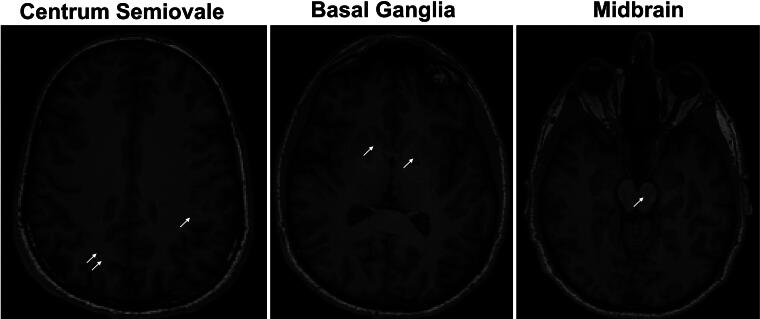
Representative slices for 3 areas of interest: centrum semiovale (left), basal ganglia (middle), and midbrain (right). Example of hypointense EPVs indicated by a white arrow. EPVs, enlarged perivascular spaces.

EPV burden was characterized using the T1 and FLAIR sequences as previously validated.^19^ Hypointense EPVs (<3 mm) were counted from representative slices in the centrum semiovale (CSO), basal ganglia, and midbrain (Fig. 1) based on the guide developed by Potter, Morris, and Wardlow.^5^ EPV counts were classified as a continuous variable (total number of hypointense lesions) and a categorical variable. For the midbrain, categories were 0 (no visible EPVs) or 1 (visible EPVs). For the basal ganglia and CSO, categories were 0 (no visible EPVs), 1 (1–10 EPVs), 2 (11–20 EPVs), 3 (21–40 EPVs), or 4 (>40 EPVs).^5^ Each scan was independently scored by two board certified neurologists (J.Y., K.S., and D.K.S.) who were blinded to ID and timepoint. If there was a disagreement in the assigned category between raters, the scan was reviewed, and a final score was decided by consensus approach. The interrater reliability for continuous data was intraclass correlation coefficient (ICC) = 0.744 and for categorical data was kappa = 0.349, which is consistent with other studies using similar methods.^22^

### Blood biomarkers

Whole blood (15 mL) was collected and processed into serum aliquots by trained clinical staff on day of enrollment (Day 1), 2 weeks postinjury, and 6 months postinjury. The Quanterix Simoa HD-X platform (Quanterix Corporation, Lexington, MA) was used to determine concentrations of neurofilament light (NfL) and GFAP using the Neurology 4-Plex B kit (Quanterix Corporation).

### Outcome measures

A battery of outcome assessments were administered at 2 weeks and 6 months postinjury to assess different domains, including functional status (Glasgow Outcome Scale-Extended [GOS-E]),^23,24^ self-reported symptomology (Rivermead Post-Concussion Symptoms Questionnaire [RPQ],^25^ Brief Symptom Inventory [BSI-18],^26^ Satisfaction with Life Survey [SWLS]^27^) and sleep [Insomnia Severity Index (ISI)^28^ and sleep subquestions of the RPQ).

### Statistical analysis

All statistical analyses were completed using STATA SE Version 16 (College Station, TX), and significance was set *a priori* as a two-sided *p* < 0.05. Examination of variable distribution for normality was conducted prior to statistical analysis choice. Biomarker measurements were natural log (ln2) transformed for analyses. GOS-E scores were dichotomized into complete (GOS-E = 8) versus incomplete (GOS-E < 8) recovery groups for analyses. To assess change over time in both EPVs and in biomarker levels, Wilcoxon signed-rank test was run. To assess the association of EPVs and outcomes, either linear (BSI-18, ISI, RPQ) or logistic (GOS-E) unadjusted regressions were performed, and correction for multiple comparisons was applied (Bonferroni, *p* < 0.003). We additionally examined the relationship between EPVs and biomarker levels using Spearman’s correlations.

## Results

A total of 32 participants with TBI were included in analyses, and demographic information is presented in Table 1. The total number of EPVs (count) at 2 weeks did not differ by the demographic variables of age, sex, head computed tomography status (normal or abnormal), and vascular risk factors (history of diabetes, hypertension, or smoking; Table 2).

**Table 1.** Demographic Information for Individuals with TBI (*N* = 32)

Age, mean ± SD	36.8 ± 13.8	
Sex, *n*(%)		
Male	25 (78%)	
Race, *n*(%)		
White	16 (50%)	
Black	11 (34%)	
Other	5 (16%)	
Education level, *n*(%)		
More than high school	23 (77%)	
High school/GED/equivalent	5 (17%)	
Less than high school	2 (7%)	
Baseline Glasgow Coma Scale Score, median [25th percentile–75th percentile]	15.0 [14.0–15.0]	
Injury cause, *n*(%)		
Road traffic incident	19 (61%)	
Fall	7 (23%)	
Other	5 (16%)	
Head CT findings, *n*(%)		
Abnormal	19 (59%)	
Loss of consciousness, *n*(%)		
Yes	19 (68%)	
Post-traumatic amnesia, *n*(%)		
Yes	17 (68%)	
Prior TBI, *n*(%)		
Yes	3 (16%)	
History of depression, *n*(%)		
Yes	4 (22%)	
History of diabetes, *n*(%)		
Yes	0 (0%)	
History of hypertension, *n*(%)		
Yes	2 (8%)	

CT, computed tomography; TBI, traumatic brain injury; SD, standard deviation.

**Table 2.** EPVs Count Among Different Demographic Factors

	2 weeks EPVs count β [95% confidence interval]
Age	0.65 [−0.01, 1.31]
Sex (male vs. female)	11.09 [−11.70, 33.88]
Head CT status (positive vs. negative)	−4.43 [−23.86, 15.00]
Vascular risk factors (history of diabetes, hypertension, and/or smoking)	3.61 [−16.51, 23.72]

CT, computed tomography; EPVs, enlarged perivascular spaces.

**FIG. 2.**
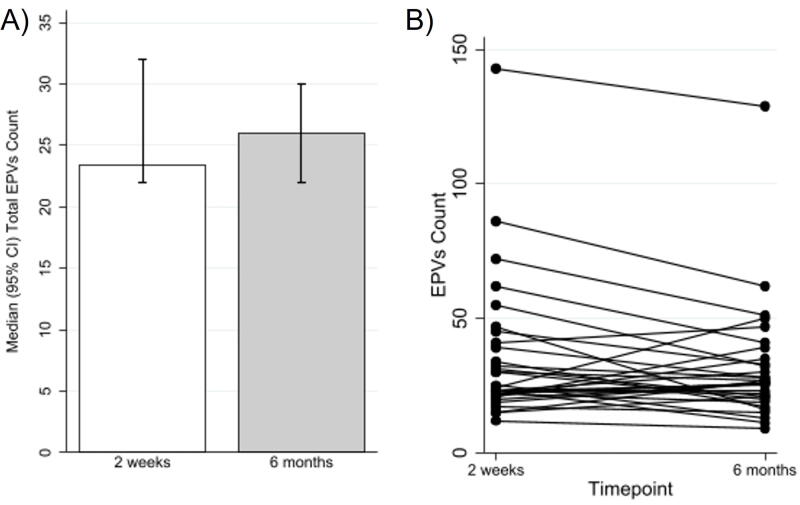
Change over time in EPVs from 2 weeks to 6 months: **(A)** for median total EPVs count (*p* = 0.16); **(B)** spaghetti plot showing each individuals trajectory from EPVs at 2 weeks to 6 months. EPVs, enlarged perivascular spaces.

**FIG. 3.**
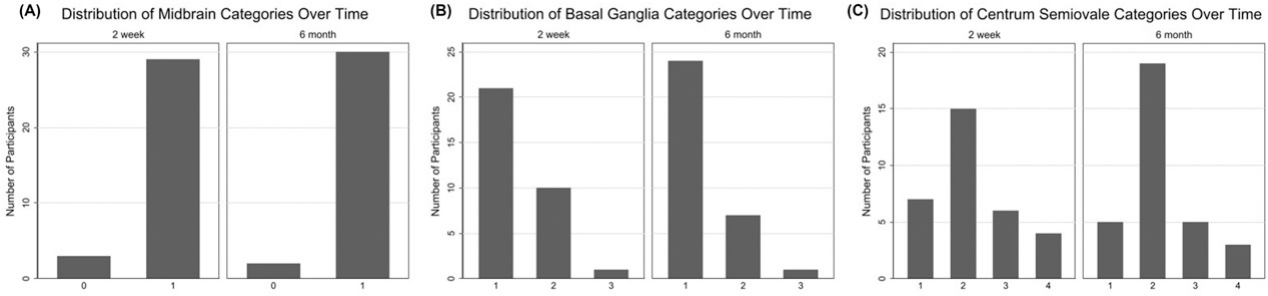
Distribution of category ratings at 2 weeks and 6 months: **(A)** midbrain, categories of 0 (no visible EPVs) or 1 (visible EPVs; *p* > 0.05); **(B)** basal ganglia, categories of 0 (no visible EPVs), 1 (1–10 EPVs), 2 (11–20 EPVs), 3 (21–40 EPVs), or 4 (>40 EPVs; *p* > 0.05); and **(C)** centrum semiovale, categories of 0 (no visible EPVs), 1 (1–10 EPVs), 2 (11–20 EPVs), 3 (21–40 EPVs), or 4 (>40 EPVs; *p* > 0.05). EPVs, enlarged perivascular spaces.

EPVs count did not significantly change from the 2-week scan (median 23.5 [95% confidence interval or CI = 22.0–32.0]) to the 6-month scan (median 26.0 [95% CI = 22.0–30.0]; *p* = 0.16; Fig. 2A). On an individual level, there was no clear pattern in change over time with some individuals increasing in EPVs and some decreasing (Fig. 2B). When examining each slice, categories of EPVs for midbrain (Fig. 3A), basal ganglia (Fig. 3B), and CSO (Fig. 3C) did not significantly change over time.

For self-reported measures of sleep, linear regressions revealed no significant associations between EPVs count and ISI or the subset of sleep questions on the RPQ (Table 3). For other self-reported symptom measures, there was a significant association for EPVs count at 2 weeks and SWLS total score at 2 weeks (β = −0.11, 95% CI = −0.18, −0.04). There was also a significant association between EPVs count at 6 months and RPQ total score measured at 6 months (β = −0.39, 95% CI = −0.66, −0.12; Table 3). The burden of basal ganglia EPVs by category (0–4) at 2 weeks and 6 months was also significantly associated with SWLS total score measured at the same timepoint (2 weeks: β = −3.93, 95% CI = −7.51, −0.35; 6 months: β = −4.92, 95% CI = −9.29, −0.54).

**Table 3.** Linear Regression Results for Association Between EPVs Count and Symptomatology Outcomes

	2 weeks EPVs count			6 months EPVs count	
	β	95% CI	β	95% CI	
ISI				
2 weeks	−0.004	−0.094, .086		
6 months	0.002	−0.122, .125	−0.137	−0.314, 0.039
RPQ sleep subquestions				
2 weeks	−0.005	−0.049, .039		
6 months	−0.019	−0.079, .042	−0.081	−0.163, 0.002
RPQ				
2 weeks	−0.094	−0.263, 0.075		
6 months	−0.143	−0.348, 0.063	**−0.385**	**−0.654, −0.117**
BSI-18				
2 weeks	−0.005	−0.105, 0.096		
6 months	0.008	−0.116, 0.132	0.047	−0.135, 0.228
SWLS				
2 weeks	**−0.112**	**−0.180, −0.044**		
6 months	−0.068	−0.171, 0.034	−0.117	−0.263, 0.029

Bolded *p* < 0.05, uncorrected.

^*^ *p* < 0.003, with Bonferroni correction.

BSI-18, Brief Symptom Inventory; CI, confidence interval; EPVs, enlarged perivascular spaces; ISI, insomnia severity index; RPQ, Rivermead Post-Concussion Symptoms Questionnaire; SWLS, Satisfaction with Life Survey.

Using logistic regression models, a higher number of total EPVs at 2 weeks were significantly associated with incomplete recovery at 6 months (GOS-E < 8) versus complete recovery (GOS-E = 8; odds ratio [OR] = 0.94, 95% CI = 0.88–0.99). There was no association between total EPVs count measured at 6 months and GOS-E measured at 6 months (GOS-E < 8 vs. GOS-E = 8; OR = 0.96, 95% CI = 0.90–1.0). By individual slice, there was a significant association with 2 weeks CSO category (OR = 0.29, 95% CI = 0.10–0.87) with GOS-E incomplete versus complete recovery where those in the lower categories of EPVs in the CSO were less likely to have complete recovery at 6 months. There were no significant associations with 2 weeks basal ganglia (OR = 0.5, 95% CI = 0.10–2.53) or midbrain (OR = 1.0, 95% CI = 0.08–12.56) EPVs with recovery measured using the GOS-E.

**FIG. 4.**
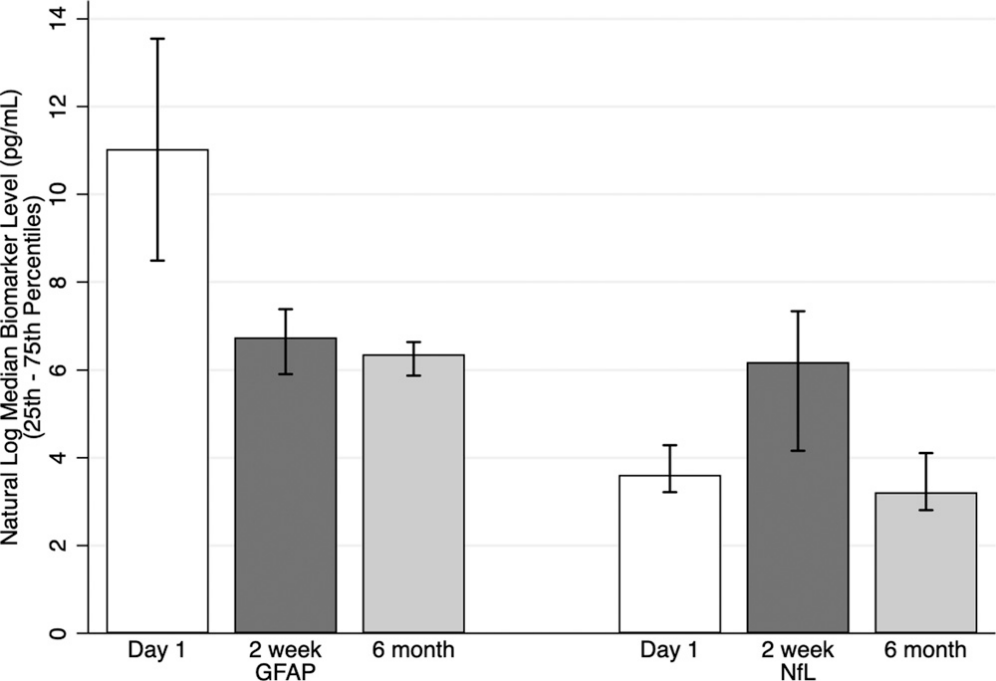
Natural log transformed median levels of GFAP (left) and NfL (right) across timepoints. * indicates *p* < 0.05 by Wilcoxon signed-rank test compared with Day 1. GFAP, glial fibrillary acidic protein; NfL, neurofilament light.

**FIG. 5.**
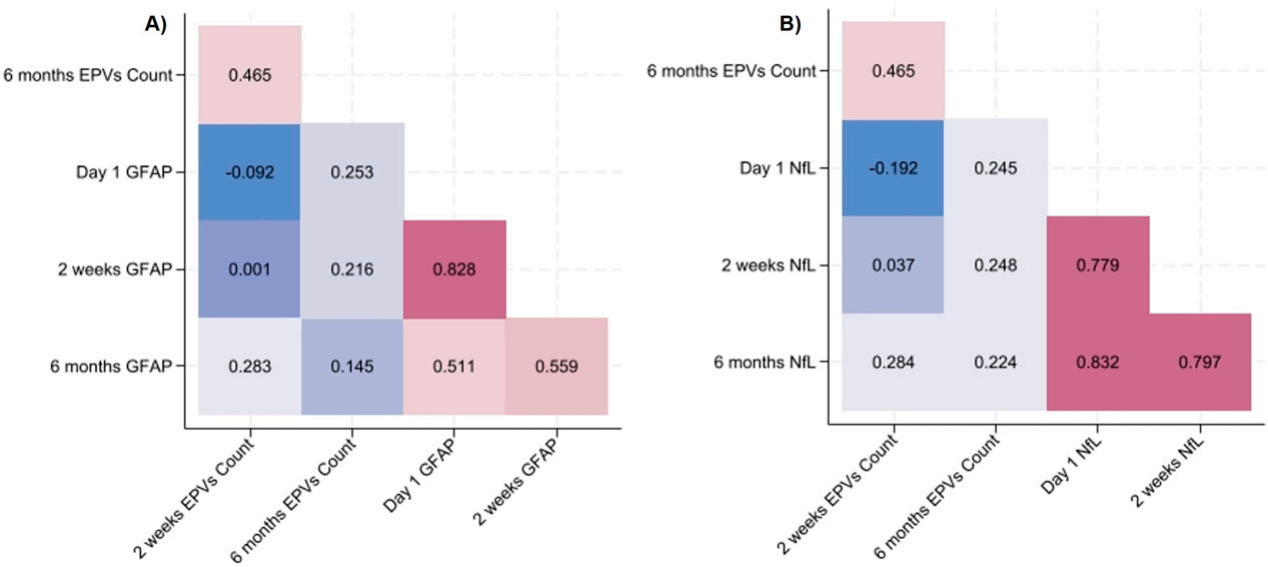
Spearman correlation matrices for EPVs count and GFAP **(A)** and NfL **(B)**. Warm colors indicate positive relationships, and cool colors indicate negative relationships. All *p*-values corrected >0.05.

Levels of circulating biomarkers GFAP and NfL were examined on Day 1, 2 weeks, and 6 months postinjury (Fig. 4). GFAP was highest on Day 1 postinjury and significantly decreased by 2 weeks and 6 months, whereas NfL peaked at 2 weeks postinjury. Spearman correlations showed no significant relationship between EPVs count and GFAP (Fig. 5A) or NfL (Fig. 5B) at any timepoint.

## Discussion

In this study, we examined the MRI burden on EPVs over time following TBI and their relationship with postinjury sleep, functional, and symptom-related outcomes. Results from this study demonstrated no change in EPVs from 2 weeks to 6 months post-TBI. This overall stability results from some individuals with increasing in EPVs, some decreasing, and most remaining relatively stable. This 6-month period of time may not be long enough for potential recovery of EPVs, or it may indicate that EPVs are permanent structural findings that do not change over time. In addition, with no MRI imaging done before TBI, we cannot determine if TBI causes EPVs or if EPVs existed prior to the injury occurring. Several other studies within a similar timeframe following TBI have shown variable ability to detect changes using different imaging modalities.^29–32^ Future work should aim at expanding the timescale and incorporating multimodal image analysis postinjury to develop a more comprehensive understanding of changes in brain structure and function following TBI.

EPVs in our cohort were heavily localized to the CSO. Many tracts run through the CSO, including the projection, commissural, and association fibers, which have implications in sensory-motor functioning.^33^ Anatomically, the CSO is adjacent to the corpus callosum, which is one of the most commonly affected brain regions in TBI as shown by advanced MRI studies.^34^ The high concentration of EPVs within the CSO is consistent with global and nonspecific white matter damage seen after TBI. Studies also hypothesize that EPVs in the CSO may be associated with vascular dysfunction,^35^ another mechanism implicated in the pathobiology of TBI, but the exact pathological correlate of EPVs remains unclear.

There was a lack of relationship between EPVs and postinjury sleep-related outcomes and measures of self-reported symptomatology in this cohort. Given that TBI is highly heterogeneous, it is not uncommon to have mixed findings when examining imaging findings with cognitive and functional outcomes. Other imaging studies, using structural or diffusion tensor imaging sequences, have also reported mixed results linking imaging metrics to outcomes.^30,36–38^ Studies linking EPVs with sleep metrics in TBI have used polysomnography^12^ and the Pittsburg Sleep Quality Inventory.^15^ These studies were conducted in military populations who had sustained their TBI(s) years before study enrollment. Military veterans are more likely to sustain recurrent and blast-related head traumas than our civilian population, which may contribute to our different results. Our study was limited by the use of self-reported sleep measures, which may not be sensitive enough to detect changes that correspond to EPV burden. More rigorous and objective sleep measures may be necessary to better evaluate dysfunctional sleep patterns following TBI.

Of the measures of self-reported symptomatology used in this study, there was minimal relationship with EPVs after correction for multiple comparisons. Only the functional outcome measure GOS-E at 6 months was significantly associated with EPVs in our cohort. GOS-E is reflective of a more global outcome after injury and does not reveal nuanced information about postinjury deficits. This relationship should be further studied as assessing EPVs on MRI is more clinically accessible (given the frequency and standard nature in which structural sequences are collected) than other more advanced MRI sequences of white matter damage, such as diffusion tensor imaging. If the relationship between EPVs and GOS-E (incomplete versus complete recovery) is confirmed in a larger study, these findings may offer clinical utility in identifying individuals likely to have an incomplete recovery earlier in their recovery period. This information would be helpful for optimizing enrollment in future therapeutic trials to those more likely to have incomplete recovery following TBI.

There was no relationship between protein brain injury biomarkers measured in the blood and EPVs in this cohort. NfL is thought to be a marker of axonal damage, whereas GFAP is an astroglia marker present in both gray and white matter.^39^ Typically, after mild TBI GFAP peaks early (Day 1) and decreases over time postinjury, whereas NfL peaks around 2 weeks;^40^ findings which are consistent with the data from our cohort. It has been suggested that GFAP, among other biomarkers, exits the brain via the glymphatic system after damage.^17^ However, other work indicates that these biomarkers enter the brain through blood–brain barrier openings.^41–43^ As such, we hypothesized that GFAP would be associated with EPVs burden; however, our data do not show a correlation between GFAP measured in the peripheral blood and the number of EPVs detected on MRI. This may be due to the way GFAP circulates in brain and is released from glial injury at the time of insult, as well as the timing of the measurements postinjury.

We used 3T MRI sequences to quantify EPVs after TBI. Although T2 sequences have been considered the gold standard,^5^ results from this study confirm that the method adapted for T1 and FLAIR sequences by Schwartz and colleagues^44^ is feasible in individuals with TBI. This could allow for quantification of EPVs burden across different neurological diseases and MRI sequences collected in both clinical and research settings where T2 sequences may be unavailable or not routinely collected, further informing future studies using data repositories from ongoing observational trials to examine these relationships in larger, longitudinally assessed cohorts.

This study is limited by a small sample size and limited timepoints following injury. In addition, our measures of sleep were based on self-report survey measures, which may not identify all sleep disturbances related to TBI. Future work should aim to examine EPVs in larger samples across longer follow-up postinjury, as well as investigating associations of EPVs burden with other MRI metrics of white matter damage, such as DTI metrics of fractional anisotropy or mean diffusivity. In addition, given the interrater reliability values here, automated methods of EPVs detection^45^ may be useful for increasing clinical utility and improving precision of EPVs detection. In addition, it is important to note that while the categorical rating method used here is standard in the literature, it draws from other neurological disease states and may not be most appropriate for use in TBI. Cohort studies with serial neuroimaging and phenotyping, along with newer automated imaging methodologies, could be combined and analyzed to better examine EPVs after TBI and their involvement in patterns of recovery.

## Conclusions

This study used 3T MRI sequences to examine the relationship between EPVs and TBI outcomes over 6 months of follow-up. Overall findings revealed that EPVs burden does not change over time after TBI. Higher EPVs at 2 weeks after injury were associated with incomplete recovery at 6 months using a global recovery measure. Further work should continue to investigate the significance of EPVs after TBI to gain better insights into whether this common radiological finding can be used as an imaging biomarker and how EPVs relate to both the underlying pathology, as well as the role in symptomology and clinical recovery following TBI.

## Transparency, Rigor, and Reproducibility Statement

This analysis plan was not formally preregistered. The first and senior author confirms that the analysis plan was prespecified. This is a secondary analysis of a larger study where only individuals with paired MRI at 2 weeks and 6 months postinjury were included resulting in 32 individuals being included. Participants were told results of clinically significant imaging findings. Clinical outcomes were assessed by team members blinded to imaging results. Analysis of imaging data was independently scored by board certified neurologists who were blinded to participant and timepoint. If there was a disagreement in the assigned category between raters, the scan was reviewed, and a final score was decided by consensus approach. MRI data were acquired on a 3T Siemens PrismaFit using a 32-channel head coil, and all data were acquired on the same scanner. Board certified neurologists performed the reads of the MRIs. The key inclusion criteria and outcome evaluations are established clinical standards. Corrections for multiple comparison were done using Bonferroni correction. No replication or external validation studies have been performed or are planned/ongoing at this time to our knowledge. The data are available from the senior author upon reasonable request. There is no MRI analytic code associated with this study. Upon publication, this article will be available in the Neurotrauma Reports.

## Compliance with Ethical Standards

All procedures involving human participants were done in accordance with the ethical standards of the institution and with the Declaration of Helsinki. Informed consent was obtained from all participants involved in this study.

## Abbreviations Used

BSI-18: Brief Symptom Inventory
CI: confidence interval
CSO: centrum semiovale
DTI: diffusion tensor imaging
EPVS: enlarged perivascular spaces
GFAP: glial fibrillary acidic protein
GOAT: Galveston Orientation and Amnesia Test
GOS-E: Glasgow Outcome Scale-Extended
ICC: intraclass correlation coefficient
ISI: Insomnia Severity Index
mL: milliliter
MRI: magnetic resonance imaging
NfL: neurofilament light
OR: odds ratio
RPQ: Rivermead Post-Concussion Symptoms Questionnaire
SWLS: Satisfaction with Life Survey
T: Tesla
TA: acquisition time
TBI: traumatic brain injury
TE: echo time
TR: repetition time

## References

[1] Braffman BH, Zimmerman RA, Trojanowski JQ, et al. Brain MR: Pathologic correlation with gross and histopathology. 1. Lacunar infarction and Virchow-Robin spaces. AJR Am J Roentgenol 1988;151(3):551–558; doi: 10.2214/ajr.151.3.551 3261517

[2] Boespflug EL, Iliff JJ. The emerging relationship between interstitial fluid-cerebrospinal fluid exchange, amyloid-β, and sleep. Biol Psychiatry 2018;83(4):328–336; doi: 10.1016/j.biopsych.2017.11.031 29279202 PMC5767516

[3] Xie L, Kang H, Xu Q, et al. Sleep drives metabolite clearance from the adult brain. Science 2013;342(6156):373–377; doi: 10.1126/science.1241224 24136970 PMC3880190

[4] Heier LA, Bauer CJ, Schwartz L, et al. Large Virchow-Robin spaces: MR-clinical correlation. AJNR Am J Neuroradiol 1989;10(5):929–936. 2505536 PMC8335297

[5] Potter GM, Chappell FM, Morris Z, et al. Cerebral perivascular spaces visible on magnetic resonance imaging: Development of a qualitative rating scale and its observer reliability. Cerebrovasc Dis 2015;39(3–4):224–231; doi: 10.1159/000375153 25823458 PMC4386144

[6] Maclullich AMJ, Wardlaw JM, Ferguson KJ, et al. Enlarged perivascular spaces are associated with cognitive function in healthy elderly men. J Neurol Neurosurg Psychiatry 2004;75(11):1519–1523; doi: 10.1136/jnnp.2003.030858 15489380 PMC1738797

[7] Park YW, Shin N-Y, Chung SJ, et al. Magnetic resonance imaging-visible perivascular spaces in basal ganglia predict cognitive decline in Parkinson’s disease. Mov Disord 2019;34(11):1672–1679; doi: 10.1002/mds.27798 31322758

[8] Wuerfel J, Haertle M, Waiczies H, et al. Perivascular spaces–MRI marker of inflammatory activity in the brain? Brain 2008;131(Pt 9):2332–2340; doi: 10.1093/brain/awn171 18676439

[9] Gertje EC, van Westen D, Panizo C, et al. Association of enlarged perivascular spaces and measures of small vessel and Alzheimer disease. Neurology 2021;96(2):e193–e202; doi: 10.1212/WNL.0000000000011046 33046608

[10] Lau K-K, Li L, Lovelock CE, et al. Clinical correlates, ethnic differences, and prognostic implications of perivascular spaces in transient ischemic attack and ischemic stroke. Stroke 2017;48(6):1470–1477; doi: 10.1161/STROKEAHA.117.016694 28495831 PMC5436733

[11] Gutierrez GM, Conte C, Lightbourne K. The relationship between impact force, neck strength, and neurocognitive performance in soccer heading in adolescent females. Pediatr Exerc Sci 2014;26(1):33–40; doi: 10.1123/pes.2013-0102 24091298

[12] Opel RA, Christy A, Boespflug EL, et al. Effects of traumatic brain injury on sleep and enlarged perivascular spaces. J Cereb Blood Flow Metab 2019;39(11):2258–2267; doi: 10.1177/0271678X18791632 30092696 PMC6827121

[13] Piantino J, Lim MM, Newgard CD, et al. Linking traumatic brain injury, sleep disruption and post-traumatic headache: A potential role for glymphatic pathway dysfunction. Curr Pain Headache Rep 2019;23(9):62; doi: 10.1007/s11916-019-0799-4 31359173

[14] Peters ME, Lyketsos CG. The glymphatic system’s role in traumatic brain injury-related neurodegeneration. Mol Psychiatry 2023;28(7):2707–2715; doi: 10.1038/s41380-023-02070-7 37185960

[15] Piantino J, Schwartz DL, Luther M, et al. Link between mild traumatic brain injury, poor sleep, and magnetic resonance imaging: Visible perivascular spaces in veterans. J Neurotrauma 2021;38(17):2391–2399; doi: 10.1089/neu.2020.7447 33599176 PMC8390772

[16] Hicks AJ, Sinclair B, Shultz SR, et al. Associations of enlarged perivascular spaces with brain lesions, brain age, and clinical outcomes in chronic traumatic brain injury. Neurology 2023;101(1):e63–e73; doi: 10.1212/WNL.0000000000207370 37156615 PMC10351302

[17] Plog BA, Dashnaw ML, Hitomi E, et al. Biomarkers of traumatic injury are transported from brain to blood via the glymphatic system. J Neurosci 2015;35(2):518–526; doi: 10.1523/JNEUROSCI.3742-14.2015 25589747 PMC4293408

[18] Huang L, Nakamura Y, Lo EH, et al. Astrocyte signaling in the neurovascular unit after central nervous system injury. Int J Mol Sci 2019;20(2):282; doi: 10.3390/ijms20020282 30642007 PMC6358919

[19] Paradise MB, Beaudoin MS, Dawes L, et al. Development and validation of a rating scale for perivascular spaces on 3T MRI. J Neurol Sci 2020;409:116621; doi: 10.1016/j.jns.2019.116621 31945583

[20] Mild traumatic brain injury committee a. Definition OF MILD TRAUMATIC BRAIN INJURY. J Head Trauma Rehabil 1993;8(3):86–87.

[21] Levin HS, O’Donnell VM, Grossman RG. The Galveston Orientation and Amnesia Test. A practical scale to assess cognition after head injury. J Nerv Ment Dis 1979;167(11):675–684; doi: 10.1097/00005053-197911000-00004 501342

[22] Bezerra DC, Sharrett AR, Matsushita K, et al. Risk factors for lacune subtypes in the Atherosclerosis Risk in Communities (ARIC) Study. Neurology 2012;78(2):102–108; doi: 10.1212/WNL.0b013e31823efc42 22170882 PMC3466671

[23] Jennett B, Bond M. Assessment of outcome after severe brain damage. Lancet 1975;1(7905):480–484; doi: 10.1016/s0140-6736(75)92830-5 46957

[24] Wilson JT, Pettigrew LE, Teasdale GM. Structured interviews for the Glasgow Outcome Scale and the Extended Glasgow Outcome Scale: Guidelines for their use. J Neurotrauma 1998;15(8):573–585; doi: 10.1089/neu.1998.15.573 9726257

[25] King NS, Crawford S, Wenden FJ, et al. The Rivermead Post-Concussion Symptoms questionnaire: A measure of symptoms commonly experienced after head injury and its reliability. J Neurol 1995;242(9):587–592; doi: 10.1007/BF00868811 8551320

[26] Derogatis LR. BSI 18, Brief Symptom Inventory 18: Administration, Scoring and Procedures Manual. NCS Pearson, Incorporated; 2001.

[27] Diener E, Emmons RA, Larsen RJ, et al. The satisfaction with life scale. J Pers Assess 1985;49(1):71–75; doi: 10.1207/s15327752jpa4901_13 16367493

[28] Bastien CH, Vallières A, Morin CM. Validation of the Insomnia severity index as an outcome measure for insomnia research. Sleep Med 2001;2(4):297–307; doi: 10.1016/s1389-9457(00)00065-4 11438246

[29] Kim E, Yoo R-E, Seong MY, et al. A systematic review and data synthesis of longitudinal changes in white matter integrity after mild traumatic brain injury assessed by diffusion tensor imaging in adults. Eur J Radiol 2022;147:110117; doi: 10.1016/j.ejrad.2021.110117 34973540

[30] Asken BM, DeKosky ST, Clugston JR, et al. Diffusion Tensor Imaging (DTI) findings in adult civilian, military, and sport-related mild traumatic brain injury (mTBI): A systematic critical review. Brain Imaging Behav 2017;12(2):585–612; doi: 10.1007/s11682-017-9708-9 28337734

[31] Mazaharally M, Stojanovski S, Trossman R, et al. Patterns of change in cortical morphometry following traumatic brain injury in adults. Hum Brain Mapp 2022;43(6):1882–1894; doi: 10.1002/hbm.25761 34953011 PMC8933328

[32] Zhuo J, Jiang L, Sours Rhodes C, et al. Early stage longitudinal subcortical volumetric changes following mild traumatic brain injury. Brain Inj 2021;35(6):725–733; doi: 10.1080/02699052.2021.1906445 33822686 PMC8207827

[33] Kourtidou P, McCauley SR, Bigler ED, et al. Centrum semiovale and corpus callosum integrity in relation to information processing speed in patients with severe traumatic brain injury. J Head Trauma Rehabil 2013;28(6):433–441; doi: 10.1097/HTR.0b013e3182585d06ED 22832369

[34] Aoki Y, Inokuchi R, Gunshin M, et al. Diffusion tensor imaging studies of mild traumatic brain injury: A meta-analysis. J Neurol Neurosurg Psychiatry 2012;83(9):870–876; doi: 10.1136/jnnp-2012-302742 22797288 PMC3415311

[35] Charidimou A, Meegahage R, Fox Z, et al. Enlarged perivascular spaces as a marker of underlying arteriopathy in intracerebral haemorrhage: A multicentre MRI cohort study. J Neurol Neurosurg Psychiatry 2013;84(6):624–629; doi: 10.1136/jnnp-2012-304434 23412074 PMC3905629

[36] Hulkower MB, Poliak DB, Rosenbaum SB, et al. A decade of DTI in traumatic brain injury: 10 years and 100 articles later. AJNR Am J Neuroradiol 2013;34(11):2064–2074; doi: 10.3174/ajnr.A3395 23306011 PMC7964847

[37] Eierud C, Craddock RC, Fletcher S, et al. Neuroimaging after mild traumatic brain injury: Review and meta-analysis. Neuroimage Clin 2014;4:283–294. 25061565 10.1016/j.nicl.2013.12.009 PMC4107372

[38] Lippa SM, Kenney K, Riedy G, et al. White matter hyperintensities are not related to symptomatology or cognitive functioning in service members with a remote history of traumatic brain injury. Neurotrauma Rep 2021;2(1):245–254; doi: 10.1089/neur.2021.0002 34223555 PMC8244514

[39] Yoon H, Walters G, Paulsen AR, et al. Astrocyte heterogeneity across the brain and spinal cord occurs developmentally, in adulthood and in response to demyelination. PLoS One 2017;12(7):e0180697; doi: 10.1371/journal.pone.0180697 28700615 PMC5507262

[40] Newcombe VFJ, Ashton NJ, Posti JP, et al. Post-acute blood biomarkers and disease progression in traumatic brain injury. Brain 2022;145(6):2064–2076; doi: 10.1093/brain/awac126 35377407 PMC9326940

[41] Dvorak F, Haberer I, Sitzer M, et al. Characterisation of the diagnostic window of serum glial fibrillary acidic protein for the differentiation of intracerebral haemorrhage and ischaemic stroke. Cerebrovasc Dis 2009;27(1):37–41; doi: 10.1159/000172632 19018136

[42] Brunkhorst R, Pfeilschifter W, Foerch C. Astroglial proteins as diagnostic markers of acute intracerebral hemorrhage-pathophysiological background and clinical findings. Transl Stroke Res 2010;1(4):246–251; doi: 10.1007/s12975-010-0040-6 24323552

[43] Mondello S, Muller U, Jeromin A, et al. Blood-based diagnostics of traumatic brain injuries. Expert Rev Mol Diagn 2011;11(1):65–78; doi: 10.1586/erm.10.104 21171922 PMC3063529

[44] Schwartz DL, Boespflug EL, Lahna DL, et al. Autoidentification of perivascular spaces in white matter using clinical field strength T1 and FLAIR MR imaging. Neuroimage 2019;202:116126; doi: 10.1016/j.neuroimage.2019.116126 31461676 PMC6819269

[45] Pham W, Lynch M, Spitz G, et al. A critical guide to the automated quantification of perivascular spaces in magnetic resonance imaging. Front Neurosci 2022;16:1021311; doi: 10.3389/fnins.2022.1021311 36590285 PMC9795229

